# Do workplace health-promotion interventions targeting employees with poor health reduce sick-leave probability and disability rates?

**DOI:** 10.1177/1403494820946543

**Published:** 2020-08-17

**Authors:** Roy A. Nielsen, Tove I. Midtsundstad

**Affiliations:** Fafo Institute for Labour and Social Research, Norway

**Keywords:** Workplace health promotion, sick leave, disability, interventions

## Abstract

**Aims:**

This study aimed to investigate whether introducing workplace health-promotion interventions targeting employees with health problems or reduced work ability affected overall sick leave and disability risk.

**Methods:**

The study population comprised data from an establishment survey from 2010 identifying who had introduced workplace health promotion (the intervention) linked to register data on all employees and their sickness absence and disability pension uptake from 2000 through 2010.

**Results:**

Interventions had moderate effects due to varying efficacy in different parts of the labour market. Intervention success was more likely among white-collar workers (e.g. in public administration) compared to blue-collar workers (e.g. in manufacturing), probably due to variations in both organisational and technological constraints. Effects were small among men and moderate among older workers, particularly among women. Overall, disability risk reduction was accompanied by an increase in sickness absence. Sometimes, sickness absence increased in groups with no change in disability risk, suggesting that presenteeism in one group may increase absenteeism in other groups.

**Conclusions:**

**Introducing workplace health-promotion interventions may prolong work careers in some labour-market segments. Financial incentives for Norwegian establishments to continue offering workplace health-promotion interventions may be improved, given the current financial model for disability pension and sickness benefits.**

## Introduction

Since the Norwegian government and the employer and employee organisations signed the Inclusive Working Life (IWL) agreement in 2001, reducing sickness absence and disability has been high on the political agenda. The aim of the IWL agreement during 2001–2018 was to ‘improve the working environment, help bring employees back to work, prevent and reduce absence due to illness, and prevent expulsion and early withdrawal from working life’ [1]. The aim corresponds to various definitions used for workplace health promotion (WHP), which generally claims to pay attention to more than just work-related risks and hazards, incorporating the maintenance and promotion of work ability, as well as paying attention to the setting in which health promotion is offered [2–6].

Several Norwegian establishments have signed the IWL agreement and implemented different measures to retain employees with health problems and reduced working capacity. In 2010, about four out of five Norwegian employees worked in so-called IWL establishments with special measures or interventions available [7]. The study aimed to investigate whether the interventions that establishments chose to implement as part of the IWL agreement had an effect on sickness absence and disability risk.

According to literature reviews, there is limited knowledge on the overall effects on sickness absence and disability of the different interventions actually implemented and offered by establishments [8,9]. Most studies evaluate the effect of a single specific workplace intervention on specific diseases or diagnoses using randomised controlled trials. Hence, we know a lot about which workplace interventions work for different diseases, but less about the health effects of measures that actually are offered and used by most establishments. In other words, do current establishments have an effective workplace strategy to combat early exit due to health problems and disability or not?

An earlier study based on Norwegian data investigated the effects of workplace interventions offered by companies to prevent sickness absence and health problems between 2001 and 2007 [10]. They found an overall reduction in sickness-absence risk in establishments with interventions compared to those without interventions, although the overall reduction in sickness absence could only be attributed to interventions in public administration. However, the special measures targeting employees with health problems offered by Norwegian establishments in 2005 seemed to cause a drop in disability risk among older workers in most sectors [11].

This study aimed to investigate the effect of WHP interventions actually implemented and offered by Norwegian companies between 2001 and 2010. We studied whether such interventions impact the sickness absence and disability risk of employees of all ages, not just older worker (i.e. those aged 50+ years). In other words, we investigated whether those interventions introduced by Norwegian establishments between 2001 and 2010 reduced sickness absence and disability risk among employees with poor health and reduced work capacity. We also examined whether these interventions benefited some occupational groups or labour-market segments more than others.

## Methods

### Study population

We used a linked employer–employee data set, consisting of data from a representative sample of 784 Norwegian establishments collected in 2010 and linked to individual register data covering all employees from 2000 through 2010. The 2010 survey provides information on whether the establishment was an IWL establishment, whether they offered WHP interventions to facilitate work among employees with health problems or reduced work ability, as well as information on industry and sector. Statistics Norway linked the 2010 survey data with annual register data on individual employees, providing information on sickness absence, disability, gender, educational level and age. The total data set comprised 279,926 individuals with more than 1.2 million observations.

### Variables

#### Outcomes: sickness absence and permanent work disability

We distinguished between sickness spells, or temporary work disability, and permanent work disability. The latter is a requirement to be eligible for a disability pension. In Norway, all employee sick-leave spells are reimbursed. Employers pay wage compensation for the first 16 (consecutive) days. When sick-leave spells last more than 16 days, the Norwegian Labour and Welfare Administration pay wage compensation (covering 100% of wages up to about €60,000 in 2019). Our data only included sickness absence compensated by the Norwegian Labour and Welfare Administration. Thus, our data covered all sickness spells lasting more than 16 days, and the dependent variable measured whether individuals had such spells (=1) within a calendar year or not. Full disability (=1) benefit is granted to eligible individuals with a permanent reduction in earning capacity due to illness and/or injury. In the case of debilitating injuries, disability benefit is granted almost immediately. However, it is more common to receive disability benefit after one or more periods of sick leave [12]. It is possible to receive sickness benefit for up to 52 weeks, possibly followed by a period with other benefits, for example work-assessment allowance, before receiving disability benefit. Thus, for individuals leaving the survey establishments, we allowed for a two-year delay in disability-benefit uptake in order to include individuals who received other benefits prior to receiving disability benefit. This means that for an individual who left a survey establishment in 2006, he or she was followed up through 2008 to identify disability-benefit uptake. Employees employed in more than one of the surveyed establishments were excluded from the analyses (748 individuals/5293 observations). Individuals who combined disability pension and work (i.e. were disabled when entering our data) were also excluded from the disability risk analyses (10,010 individuals/35,988 observations). Thus, we investigated whether interventions reduced the risk for new disability cases.

#### Predictor variable: introduction of WHP measures

**Figure 1.**
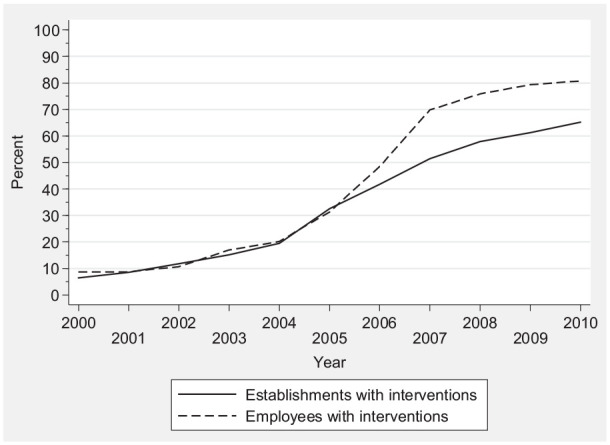
Share of establishments and employees with workplace health promotion interventions to promote longer working careers among employees with health problems and reduced work ability, 2000–2010 (%).

In the survey, the establishments’ personnel manager/human resource manager answered ‘yes’ or ‘no’ to the following question: ‘Does your establishment offer special measures to promote longer working careers among employees with health problems and reduced work ability?’ Those answering ‘yes’ where asked to specify the type of interventions offered. It was most common to offer some sort of work accommodation, such as workplace or work-task adjustments, easier and/or other work tasks, reduced working hours or ergonomic and technical assistance. All of these measures are more common in public-sector than in private-sector establishments and organisations. The survey also mapped whether the establishments had signed the IWL agreement and whether they offered retention measures targeting employees close to retirement age. If applicable, managers also declared when the agreement was signed and when they introduced their retention measures, which generally coincided with regard to timing. We used this information to date the year in which the special measures (to promote longer working careers among employees with health problems and reduced work ability) were introduced, based on the assumption that establishments signing the IWL agreement intensified their efforts to reach the goals of the IWL agreement. Previous studies assumed a major shift in IWL participation around 2004–2005 [10,11], which is corroborated here (Figure 1). However, in our view, the approach used here is more precise. To allow the introduction of WHP measures to take time before having an effect, introduction of measures was measured as time (in years) since implementation.

### Confounders

We adjusted our analyses by calendar year, age (and age squared), average age, share of females, share aged ⩾50 years and number of employees in establishments, all of which are known to be associated with levels of absence from work [6,13].

### Analyses

We estimated the effect of the WHP measures on both sickness absence and full disability using individual fixed-effects regression. Thus, we viewed the introduction of special measures as a natural (or quasi) experiment, where employees in establishments with measures were exposed, and employees in the remaining establishments were unexposed. A natural experimental approach has been recommended when using observational data to evaluate population health interventions [14], and fixed-effects regression is among the recommended study designs [15]. As a side note, some find the term ‘fixed effects’ confusing. Although it is widely used in econometrics [16,17], it is also referred to as a within-individuals estimate [18] or as ‘unobserved effects’ [19], the point being that all time-invariant variables, observed or not, are treated as fixed parameters which normally are conditioned out of the estimation [20]. The basic idea is that each individual acts as his or her own control [21], and that all time-invariant individual confounding is controlled for [22], which may reduce potential bias in causal estimates. In addition, treatment assignment, that is, access to special measures in this article, should be ‘as good as random’ [19,22,23]. The WHP measures under investigation here were introduced at the establishment level. Most employees probably did not know of the interventions beforehand, and employees were unlikely to choose (if they could) their employer based on the (future) presence of such interventions. Consequently, the distribution of exposed and unexposed employees is likely to be ‘as good as random’. On the other hand, the introduction of interventions may vary systematically by sector and industry, and employees in different industries may have different characteristics. However, if such characteristics are time invariant, they are not a problem in our applied design. As sector and industry, as well as gender and educational level, are time invariant, it is not possible to estimate their effect using fixed-effects regression. However, separate analyses of the different categories allowed us to investigate if and how the introduction of interventions impacted sick-leave absence and permanent disability between groups. We estimated linear probability models using the xtreg-procedure with fixed effects and robust standard errors. Data analyses were conducted using Stata v14.2 (StataCorp LLC, College Station, TX).

## Results

In 2000, 3% of establishments had introduced WHP interventions to reduce sickness absence and disability (Figure 1). This share increased to about 10% in 2004, and then the share of establishments with interventions doubled to 20% in 2005 and increased to 40% in 2010. The share of employees in establishments with interventions increased from about 7% in 2000 to almost 70% in 2010. Thus, establishments with a high number of employees were more likely to introduce these interventions.

In 2010, employees in establishments without WHP interventions were approximately the same age compared to those in establishments with interventions, whereas there was a higher share of females in establishments with interventions (Table I). The distribution by educational level was quite similar in establishments without or with interventions. However, there were more employees with secondary education in establishments without interventions. Thus, the share of individuals with interventions was the lowest in this group. Among establishments without interventions, two out of three were in the private sector. Among employees with interventions in place, most were working within public administration or in health and social services. However, interventions were also common within manufacturing. Interventions were rarest among employees in construction.

**Table I.** Descriptive statistics of employees in companies with and without WHP interventions to promote longer working careers among employees with health problems and reduced work ability, 2010.

	No interventions	Interventions
Establishments, *n* (%)	273 (34.8)	511 (65.2)
Employees, *n* (%)	23,593 (19.2)	99,043 (80.8)
Age, mean (*SD*)	43.0 (12.6)	43.9 (12.8)
Educational level (within level)		
Compulsory or unknown	18.0 (21.1)	16.1 (78.9)
Secondary	40.0 (20.3)	37.3 (79.7)
Lower-level university	32.5 (18.2)	34.7 (81.8)
Higher-level university	9.4 (15.9)	11.9 (84.1)
	100.0 (19.2)	100.0 (80.8)
Business sector (within sector)		
Private sector	44.0 (21.5)	38.2 (78.5)
Public sector, central and local government	56.0 (17.8)	61.8 (82.2)
	100.0 (19.2)	100.0 (80.8)
Industry (within industry)		
Manufacturing	9.0 (24.7)	6.6 (75.3)
Retail	11.5 (28.2)	6.8 (71.8)
Professional, scientific and technical activities	4.8 (26.0)	3.3 (74.0)
Public administration	29.9 (10.4)	61.6 (89.6)
Health and social services	26.7 (41.7)	8.9 (58.3)
Other industries	18.3 (25.3)	12.9 (74.8)
	100.0 (19.2)	100.0 (80.8)

WHP: workplace health promotion.

Overall, we found a moderate effect of WHP interventions on the probability of both sickness absence and permanent disability. Sickness absence increased about half a percentage point per year following the introduction of interventions among males younger than 50 years of age (however, diminishing over time), whereas there was no impact among older males (Table II). When investigating the effects within different subgroups, we found that the strongest increase in sickness absence was among males younger than 50 years of age with basic education. No male groups experienced a reduction in sickness absence following the introduction of interventions. Among females, the overall effect of measures was somewhat higher compared to males, with slightly more than one percentage point increase in sickness absence per year since the introduction among females younger than 50 years of age, and almost the same among females aged ⩾60 years. Among females aged 50–59 years, there was a slight decrease in sickness absence following the introduction of interventions. The largest impact was found among young females in health and social services when investigating differences between industries, and in the private sector when focusing on differences between sectors. Similar to what was found among men, there was an increase in sickness absence among young females with basic education. The largest decrease was found among females aged 50–59 years with basic education, many of whom probably worked in municipalities, which was the only industry (business sector) where sickness absence was reduced. Among females aged ⩾60 years, the only significant increase was within health and social services.

**Table II.** Change in probability of sickness absence (>16 days) following introduction of WHP interventions to promote longer working careers among employees with health problems and reduced work ability: linear probability model with individual fixed effects and robust standard errors.

	Males												Females											
	15–49 years				50–59 years				60+ year				15–49 years				50–59 years				60+ years			
	Time		Time×time		Time		Time×time		Time		Time×time		Time		Time×time		Time		Time×time		Time		Time×time	
Sick leave	b.	s.e.	b.	s.e.	b.	s.e.	b.	s.e.	b.	s.e.	b.	s.e.	b.	s.e.	b.	s.e.	b.	s.e.	b.	s.e.	b.	s.e.	b.	s.e.
*Overall*	**0.005**	0.002	**–0.001**	0.000	−0.003	0.003	0.000	0.000	−0.008	0.005	0.001	0.000	**0.012**	0.002	**–0.001**	0.000	**–0.004**	0.003	**0.001**	0.000	**0.010**	0.005	0.000	0.000
*Educational level*																								
Basic	**0.017**	0.005	**–0.002**	0.001	0.002	0.010	0.000	0.001	−0.014	0.018	0.001	0.002	**0.022**	0.005	**–0.002**	0.001	**–0.016**	0.008	**0.002**	0.001	0.015	0.011	−0.001	0.001
Secondary	**0.005**	0.003	**–0.001**	0.000	−0.009	0.005	0.001	0.000	−0.006	0.008	0.000	0.001	**0.009**	0.003	**–0.001**	0.000	−0.002	0.004	0.001	0.000	0.003	0.007	0.001	0.001
Tertiary I	0.002	0.003	0.000	0.000	−0.002	0.006	0.000	0.001	−0.005	0.011	0.001	0.001	**0.011**	0.003	**–0.001**	0.000	−0.006	0.005	0.001	0.001	0.013	0.010	−0.001	0.001
Tertiary II	0.000	0.003	0.000	0.000	0.006	0.006	0.000	0.001	0.000	0.011	0.000	0.001	**0.007**	0.006	**–0.001**	0.001	0.006	0.014	0.000	0.001	0.030	0.023	−0.004	0.002
*Industry*																								
Manufacturing	0.002	0.004	0.000	0.000	−0.004	0.007	0.000	0.001	0.008	0.014	−0.001	0.001	**0.014**	0.007	**–0.002**	0.001	−0.014	0.015	0.001	0.001	0.014	0.030	0.000	0.003
Retail	−0.003	0.006	0.001	0.001	−0.011	0.014	0.000	0.001	−0.034	0.019	**0.003**	0.002	0.012	0.009	−0.002	0.001	0.004	0.016	−0.003	0.002	0.024	0.025	−0.001	0.003
Professional services	0.009	0.007	−0.001	0.001	0.001	0.017	−0.002	0.002	−0.027	0.033	0.004	0.005	−0.013	0.016	0.000	0.003	0.003	0.033	−0.002	0.005	0.021	0.043	−0.006	0.007
Public administration	**0.009**	0.003	**–0.001**	0.000	0.002	0.005	0.000	0.001	−0.001	0.008	0.000	0.001	0.010	0.002	−0.001	0.000	−0.006	0.004	0.001	0.000	0.007	0.006	0.000	0.001
Health and social services	0.005	0.008	0.000	0.001	0.019	0.014	−0.001	0.002	−0.025	0.021	0.003	0.002	**0.028**	0.005	**–0.002**	0.001	−0.002	0.009	0.000	0.001	**0.022**	0.013	**–0.003**	0.001
*Business sector*																								
Central government	**0.004**	0.005	**–0.001**	0.001	**0.014**	0.008	**–0.003**	0.001	−0.006	0.013	0.001	0.001	0.010	0.005	−0.001	0.001	0.005	0.009	0.001	0.001	0.030	0.014	−0.002	0.002
Municipalities	0.002	0.005	−0.001	0.000	−0.003	0.007	0.000	0.001	−0.001	0.011	−0.001	0.001	0.003	0.003	0.000	0.000	**–0.009**	0.004	**0.001**	0.000	0.007	0.007	0.000	0.001
Private sector	**0.010**	0.002	**–0.001**	0.000	−0.008	0.005	0.001	0.000	−0.004	0.007	0.001	0.001	**0.024**	0.003	**–0.002**	0.000	0.001	0.006	0.000	0.001	0.018	0.010	−0.001	0.001

Time and time×time are measures of years since introduction of work accommodations (interventions). Significant coefficients in shown in bold. Adjusted for age (including a polynomial) calendar year dummies, establishment size, within establishment (a) average age, (b) share of females and (c) share of employees aged ⩾50 years.

Turning our attention to the risk for permanent disability, there were only minor effects from introducing WHP interventions, and most so among older employees (Table III). Among males aged ⩾60 years, there was a reduction in disability risk by about half a percentage point per year following the introduction of interventions, with the largest effect – about 1.5 percentage points per year – in central government. Among females, the interventions had a minor but significant impact among those aged 50–59 years, and the strongest effects were found among females aged ⩾60 years. Overall, the effect among females aged ⩾60 years was close to two percentage points per year, with the strongest reduction in disability risk among employees in municipalities compared to the governmental and private sector. When comparing industries, we found that the reduction was strongest in public administration and health and social services (which is part of the municipality sector), which corresponds to the finding of differences in disability reduction between sectors.

**Table III.** Change in probability of permanent work disability following introduction of WHP interventions to promote longer working careers among employees with health problems and reduced work ability. Linear probability model with individual fixed effects and robust standard errors.

	Males												Females											
	15–49 years				50–59 years				60+ years				15–49 years				50–59 years				60+ years			
	Time		Time×time		Time		Time×time		Time		Time×time		Time		Time×time		Time		Time×time		Time		Time×time	
Sick leave	b.	s.e.	b.	s.e.	b.	s.e.	b.	s.e.	b.	s.e.	b.	s.e.	b.	s.e.	b.	s.e.	b.	s.e.	b.	s.e.	b.	s.e.	b.	s.e.
*Overall*	0.000	0.000	0.000	0.000	**–0.002**	0.001	**0.000**	0.000	**–0.006**	0.003	0.000	0.000	**0.000**	0.000	0.000	0.000	**–0.006**	0.001	**0.000**	0.000	**–0.018**	0.003	**0.001**	0.000
*Educational level*																								
Basic	−0.001	0.001	0.000	0.000	−0.001	0.004	0.000	0.000	−0.003	0.009	0.000	0.001	**–0.001**	0.000	0.000	0.000	**–0.012**	0.003	0.001	0.000	**–0.020**	0.006	**0.002**	0.001
Secondary	0.000	0.000	0.000	0.000	−0.003	0.001	0.000	0.000	−0.001	0.004	0.000	0.000	**–0.001**	0.000	0.000	0.000	**–0.006**	0.001	**0.000**	0.000	**–0.018**	0.004	**0.001**	0.000
Tertiary I	0.000	0.000	0.000	0.000	**–0.005**	0.002	0.000	0.000	−0.010	0.005	0.000	0.000	0.000	0.000	0.000	0.000	**–0.005**	0.001	**0.000**	0.000	**–0.015**	0.006	0.001	0.001
Tertiary II	0.000	0.000	0.000	0.000	0.002	0.001	0.000	0.000	**–0.009**	0.004	0.001	0.000	0.000	0.000	0.000	0.000	0.002	0.003	0.000	0.000	−0.006	0.009	0.000	0.000
*Industry*																								
Manufacturing	0.000	0.000	0.000	0.000	0.001	0.003	0.000	0.000	0.002	0.007	−0.001	0.001	0.000	0.000	0.000	0.000	**–0.013**	0.005	0.000	0.000	−0.020	0.015	0.001	0.001
Retail	0.000	0.000	0.000	0.000	−0.009	0.005	0.001	0.001	0.001	0.010	0.000	0.001	−0.001	0.001	0.000	0.000	**–0.019**	0.006	**0.002**	0.001	−0.022	0.021	0.005	0.004
Professional services	0.000	0.000	0.000	0.000	−0.006	0.005	0.001	0.001	−0.013	0.010	0.001	0.001	−0.001	0.001	0.000	0.000	−0.011	0.008	0.001	0.001	−0.037	0.023	0.005	0.004
Public administration	0.000	0.000	0.000	0.000	**–0.004**	0.001	**0.000**	0.000	−0.008	0.004	0.000	0.000	**0.000**	0.000	0.000	0.000	**–0.005**	0.001	**0.000**	0.000	**–0.025**	0.004	**0.002**	0.000
Health and social services	0.000	0.001	0,000	0.000	0.004	0.004	−0.001	0.000	−0.002	0.012	0.000	0.001	0.000	0.000	0.000	0.000	**–0.005**	0.002	0.000	0.000	**–0.024**	0.007	**0.002**	0.001
*Business Sector*																								
Central government	−0.001	0.000	0.000	0.000	**–0.006**	0.002	0.001	0.000	**–0.017**	0.007	**0.002**	0.001	0.000	0.000	0.000	0.000	**–0.007**	0.002	0.000	0.000	−0.011	0.007	0.000	0.001
Municipalities	0.000	0.001	0.000	0.000	**–0.008**	0.002	**0.000**	0.000	−0.010	0.007	0.000	0.001	0.000	0.000	0.000	0.000	**–0.010**	0.001	**0.001**	0.000	**–0.031**	0.004	**0.002**	0.000
Private sector	0.000	0.000	0.000	0.000	0.001	0.002	0.000	0.000	0.000	0.004	0.000	0.000	0.000	0.000	0.000	0.000	**–0.004**	0.002	0.000	0.000	−0.001	0.005	0.000	0.000

Time and time×time are measures of years since introduction of WHP (interventions). Significant coefficients in shown in bold. Adjusted for age (including a polynomial) calendar year dummies, establishment size, within establishment (a) average age, (b) share of females and (c) share of employees aged ⩾50 years.

## Discussion

Among employees in establishments offering WHP measures to employees with health problems and reduced work capacity, the measures caused a moderate increase in sickness absence and a small reduction in disability risk in select groups. Female employees aged 50–59 years were the only group to experience a reduction in both sickness absence and disability risk following the introduction of interventions. Analyses by subgroups showed that this effect only holds for females aged 50–59 years with basic education employed in municipalities. Some groups (e.g. females aged ⩾60 years) experienced both a reduction in disability risk and an increase in sickness absence. Finally, in some instances (e.g. females aged 50–59 years in manufacturing), there was a reduction in disability risk and no change in sickness absence. Instead, we found an increase in sickness absence among the youngest females employed in manufacturing. Overall, the WHP interventions had no or small effects, particularly among males. Although the result may be seen as surprising, it is in line with findings in earlier Norwegian studies [10,11]. And while the results may seem minor, their implications may be very important.

One possible explanation of this somewhat surprising effect could be the conflicting goals of the Norwegian IWL agreement. The goals of the agreement are both to reduce the rate of disability pensioners and to reduce sickness absence. However, if establishment measures reduce disability rates, more employees with health problems and reduced work capacity will be retained, which in turn may increase sickness absence.

In line with this, we found the effects of WHP interventions on sickness absence to be strongest among females aged ⩾60 years, who also experienced the strongest decrease in disability risk. In addition, we found that interventions increased sickness-absence risk among females aged 15–49 years. This was especially the case in the health and social services sector where the risk of disability among the oldest also decreased the most. This could possibly be due to the implementation process and the typical interventions chosen by the establishments, such as reduced working hours and easier and other work tasks. These measures not only influence the workload for those with health problems and reduced working capacity, but may also have an impact on other employees’ working conditions. When someone gets easier work tasks, others have to take more of the heavier work, whether it is colleagues or middle managers [7,24]. This could happen if management fail to allocate the necessary resources, for example funds to hire substitutes. In addition, many establishments lack alternative work tasks, or they cannot offer alternative work-time arrangements for all employees in need of work adjustments [24]. Thus, if costs of interventions and adjustment of work conditions for select employees are transferred to colleagues and middle managers, they may have negative unintended side effects, and can in the end increase the likelihood of sickness absence among other employees, contrary to intentions. However, at a societal level, it is probably better that an individual is working at reduced capacity rather than only receiving benefits.

The WHP interventions offered by the establishments reduced the disability risk more among older workers compared to younger workers. An explanation might be that sickness absence due to workplace conditions in general is more common among older workers. Thus, older workers’ health problems are more often caused by work-related factors, and hence may be easier to remedy through work-related measures and adjustments.

The effects of interventions on disability risk were also more visible in public administration compared to, for example, manufacturing. This was the case for both males and females. It may be easier to adjust or reduce work tasks and/or adjust the work environment for individual employees in public administration (mostly white-collar workers) than in manufacturing (mostly blue-collar workers). Previous surveys have found that Norwegian managers in manufacturing are less willing to offer older workers reduced working hours and work adjustments than managers in the public sector are [7,25]. This is due to both organisational and technological constraints, with few alternative tasks available in many establishments, as well as establishment culture, where part-time work and reduced working hours have been less common.

Regarding differences in effects of interventions on disability risk between males and females, it may be that females in general find it easier to talk about and reveal health problems (their weaknesses) [24]. Hence, females may be more likely than males to seek special arrangements and adjustments, and consequently, they may receive more support, even if they have the same health problems and needs.

### Methodological considerations

Our study has some methodological shortcomings. The main challenge is that we do not know whether the individual employees were actually offered any measures. We only know that the establishments’ personnel managers stated that the establishment had such measures. On the other hand, as discussed earlier in this article, implementation of such measures might affect both those who get a work adjustment as well as their colleagues. What we in fact measured is therefore both the so-called intended and the so-called unintended effect of the special interventions offered to employees with health problems and reduced working capacity in order to increase their employability. Furthermore, the interventions studied here were aimed at individuals with health problems or reduced work ability. Thus, they do not (necessarily) target all employees. Unfortunately, other than the outcome variables, we did not have data on health problems or work ability among employees, although it is well known that a large share of employees report such problems. Thus, the ability to identify employees with health problems or reduced work ability and whether they were offered accommodations may yield better estimates on their efficacy.

## Conclusions

Our study found that Norwegian establishments’ WHP interventions to promote longer working careers among employees with health problems and reduced work ability affected overall sickness absence and disability risk only to a minor degree. However, this was mainly due to interventions only having an impact in parts of the labour market. Furthermore, the interventions reduced disability risk but increased sickness absence. Retaining employees with health problems may then cause an increase in sickness absence, although our study suggests that they prolong working careers for some. More studies are needed to understand why the alleviative accommodations offered only work in some parts of the labour market. Future studies should, if possible, have a longer follow-up. In addition, further dispersion of such accommodations may be dependent on additional incentives targeting employers.

The Norwegian welfare state takes the financial burden of long-term sickness absence and permanent disability, while Norwegian establishments bear the costs of short-term sickness absence (up to 16 days). Norwegian establishments also have to finance workplace interventions, despite results suggesting that it is the welfare state which will gain most financially from reduced disability rates, although interventions increase the risk of long-term sickness absence in some groups. Thus, financial incentives for Norwegian establishments to continue offering such interventions may be improved, given the current financial model for disability pension and sickness benefits.
